# Evolutionary Perspectives on the Moonlighting Functions of Bacterial Factors That Support Actin-Based Motility

**DOI:** 10.1128/mBio.01520-19

**Published:** 2019-08-27

**Authors:** Volkan K. Köseoğlu, Hervé Agaisse

**Affiliations:** a Department of Microbiology, Immunology, and Cancer Biology, University of Virginia School of Medicine, Charlottesville, Virginia, USA; University of Illinois at Chicago

**Keywords:** ActA, actin-based motility, IcsA, *Listeria*, *Shigella*, adhesion, aggregation, biofilm, extracellular pathogen, intracellular pathogen, invasion, moonlighting

## Abstract

Various bacterial pathogens display an intracellular lifestyle and spread from cell to cell through actin-based motility (ABM). ABM requires actin polymerization at the bacterial pole and is mediated by the expression of bacterial factors that hijack the host cell actin nucleation machinery or exhibit intrinsic actin nucleation properties. It is increasingly recognized that bacterial ABM factors, in addition to having a crucial task during the intracellular phase of infection, display “moonlighting” adhesin functions, such as bacterial aggregation, biofilm formation, and host cell adhesion/invasion. Here, we review our current knowledge of ABM factors and their additional functions, and we propose that intracellular ABM functions have evolved from ancestral, extracellular adhesin functions.

## Abstract (precis)

Various bacterial pathogens display an intracellular lifestyle and spread from cell to cell through actin-based motility (ABM). ABM requires actin polymerization at the bacterial pole and is mediated by the expression of bacterial factors that hijack the host cell actin nucleation machinery or exhibit intrinsic actin nucleation properties.

## PERSPECTIVE

The functions of genes are generally discovered through the identification of genetic alterations (genotype) that correlate with alterations in observable biological traits (phenotype). For reasons that may be related to the enlightening nature of uncovering the unknown, it is dogmatically accepted that genes display one, and only one, function, that is, the biological function under investigation at the time of gene discovery. However, it is becoming increasingly apparent that a given gene may encode a single protein that displays various functions in addition to its first-discovered “canonical” function. This ability of a protein to have more than one biological function is referred to as “moonlighting” (1). Diverse sets of proteins from all domains of life demonstrate moonlighting functions, and they include metabolic enzymes, transcription factors, chaperones, and ribosomal proteins (1, 2). For instance, the glycolytic enzyme glyceraldehyde-3-phosphate dehydrogenase (GAPDH) conducts alternative tasks both in eukaryotic and prokaryotic cells. On the surfaces of macrophages, GAPDH isoforms participate in the maintenance of iron homeostasis, functioning as a receptor for iron binding proteins, such as lactoferrin (3), transferrin (4), and apotransferrin (5). In addition, when localized to the nucleus, GAPDH promotes either cell death or increased cell survival (6). In bacterial pathogens, extracellular GAPDH operates as a virulence factor, contributing to bacterial adherence to host cells (7–9), to interactions between different bacterial species that facilitate host colonization (10), and to evasion from the host immune system (7, 11, 12).

**FIG 1:**
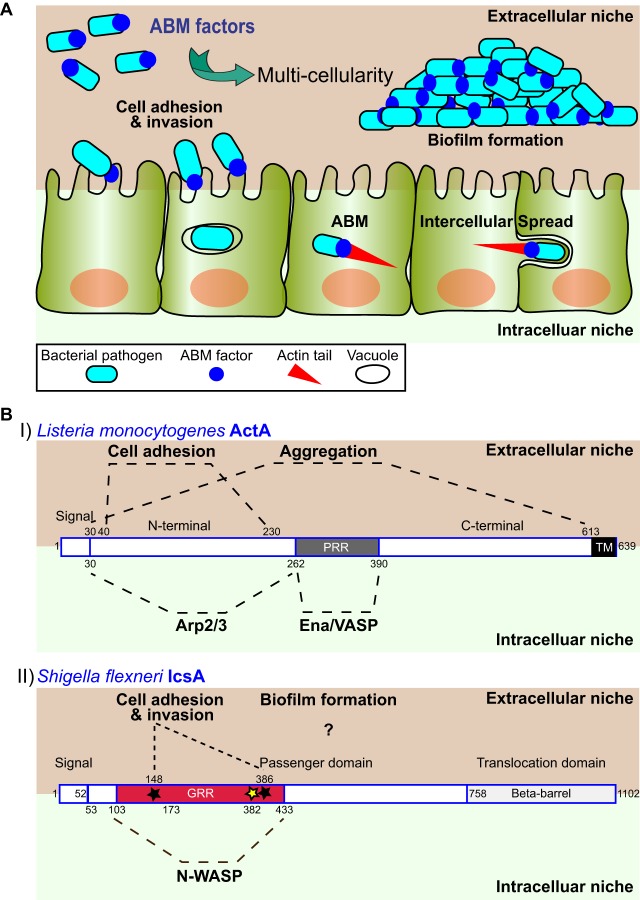
Moonlighting functions of bacterial factors that support actin-based motility (ABM). (A) In the intracellular niche (green background), ABM factors promote ABM and intercellular spread. In the extracellular niche (beige background), ABM factors promote interactions with host cells (adhesion and invasion) and biofilm formation. (B) Structural elements of ABM factors and corresponding functions. (I) Domain organization of Listeria monocytogenes ActA and structural/functional elements. The extracellular niche consists of the gut lumen, cell surface, and *in vitro* conditions. Cell adhesion is carried out by the region from amino acids 40 to 230; aggregation is carried out by full-length ActA. The intracellular niche is the host cell cytoplasm. ARP2/3 recruitment is carried out by the region from amino acids 30 to 262. Ena/VASP recruitment is carried out by the proline-rich region (PRR) from amino acids 262 to 390. TM, transmembrane domain. (II) Domain organization of Shigella flexneri IcsA and structural/functional elements. The extracellular niche is the cell surface and *in vitro* growth conditions. Cell adhesion and invasion is carried out by amino acid residues 148 and 386 (black stars); the region required for biofilm formation is unknown (question mark). The intracellular niche is the host cell cytoplasm. N-WASP recruitment is carried out by the glycine-rich repeat (GRR)-containing region (amino acids 103 to 433 and amino acid residue 382 [yellow star]). Dashed lines indicate ActA and IcsA regions and their interacting host factors (intracellular niche) or associated functions (extracellular niche). Numbers indicate amino acid residue positions.

Here, we discuss the extracellular moonlighting functions of bacterial factors that support the intracellular lifestyle of cytosolic pathogens displaying actin-based motility (ABM). Various intracellular pathogens, such as Listeria monocytogenes, Shigella flexneri, *Rickettsia* spp., and *Burkholderia* spp., reside in the cytosol of infected cells, where they acquire ABM through expression of bacterial factors that hijack the host cell actin polymerization machinery or exhibit intrinsic actin nucleation capacity (13). Expression of these ABM factors in heterologous hosts is sufficient to confer actin-based motility (14, 15). Actin polymerization at the bacterial pole generates forces that propel the pathogen throughout the cytosol (Fig. 1A). At cell-cell contacts, ABM mediates intercellular spread through the formation of membrane protrusions that resolve into vacuoles from which the pathogen escapes, thereby gaining access to the cytosolic compartment of adjacent cells (16, 17) (Fig. 1A). It has recently emerged that bacterial ABM factors, such as Listeria monocytogenes ActA and Shigella flexneri IcsA, perform extracellular moonlighting adhesin functions that promote self-aggregation and host cell adhesion, in addition to having a paramount role in intracellular ABM (Fig. 1A).

L. monocytogenes ABM relies on ActA (18), a bacterial factor that binds and activates the Arp2/3 complex, a critical host cell actin nucleator (19) (Fig. 1B, panel I). ActA is displayed at the bacterial pole, which is critical for actin polymerization and generation of forces that propel the pathogen throughout the cytosol (20). Seminal studies uncovered the ActA structural determinants that mediate ActA-Arp2/3 interaction. The actin nucleation activity of the Arp2/3 complex is stimulated by the N-terminal domain of ActA (21), which mimics the regulatory activity of the host cell nucleation-promoting factor neural Wiskott-Aldrich syndrome protein (N-WASP), leading to recruitment and activation of the ARP2/3 complex (22) (Fig. 1B, panel I, region from amino acids 30 to 262), as well as to the recruitment of additional host cell actin cytoskeleton regulators, such as Ena/VASP proteins (23) (Fig. 1B, panel I, PRR region). The C-terminal domain anchors ActA to the bacterial cell wall and is not known to interact with any actin cytoskeleton components (Fig. 1B, panel I).

Subsequent to the discovery of the intracellular role of ActA in ABM, various reports revealed that ActA mediates extracellular moonlighting functions, including adhesion to and invasion of host cells and host colonization. L. monocytogenes invades different host cell types, primarily through the internalin proteins, such as InlA and InlB, which bind host cell receptors (24). In addition to internalins, ActA was suggested to be required for epithelial cell invasion, potentially through adhesion to microvillus structures at the apical surfaces of epithelial cells (25) (Fig. 1B, panel I). In addition to invading host cells, ActA mediates L. monocytogenes aggregation *in vitro* as well as biofilm formation, through ActA-ActA self-interaction (26). Importantly, ActA-mediated aggregation was also observed *in vivo* in a mouse model of intestinal infection and facilitated persistent L. monocytogenes colonization. *In vitro* aggregation and long-term intestinal colonization require full-length ActA, and structure/function analysis revealed an aggregation-specific role for the C-terminal domain of ActA (Fig. 1B, C-terminal G394-R585 region), which is not required for ABM (26).

As with L. monocytogenes ActA, the bacterial factor supporting S. flexneri ABM, IcsA (27), exhibits extracellular moonlighting functions, including biofilm formation (28, 29) (Fig. 1A). IcsA bears the classical domain organization of type Va autotransporters, which is composed of an N-terminal signal sequence, a surface-exposed passenger domain, and the beta-barrel translocation domain (30) (Fig. 1B, panel II). As with ActA, IcsA is displayed at the bacterial pole (31, 32). Unlike ActA, which promotes the nucleation activity of the Arp2/3 complex, IcsA recruits the host cell actin nucleation-promoting factor N-WASP, which subsequently binds and activates the Arp2/3 complex (33, 34). Structure/function analyses of the IcsA passenger domain showed that the region from R103 to A433 is responsible for N-WASP binding *in vivo* and *in vitro* (35, 36) (Fig. 1B, panel II).

In addition to having a role in ABM, IcsA functions as a polar adhesin and promotes invasion upon exposure to bile salts (37). The ABM and adhesin functions of IcsA were genetically dissected. A mutant IcsA protein carrying two individual insertions with no apparent effect on ABM (36) displayed decreased adhesion and invasion upon bile salt exposure (37) (Fig. 1B, panel II, black stars). The role of bile salts was also investigated in the context of *in vitro* biofilm formation (28, 29). The structural determinant(s) supporting IcsA-mediated biofilm formation upon bile salt exposure remains to be determined. However, IcsA self-associates at the bacterial poles of individual bacteria, which is critical for N-WASP recruitment (38). Thus, one potential scenario in the context of biofilm formation is that IcsA interbacterial self-association may contribute to bacterial aggregation (29). Interestingly, IcsA shares structural similarities with autotransporter adhesins, such as Escherichia coli Ag43 (35), that mediate aggregation and biofilm formation through self-association (39). We note that what distinguishes IcsA from these adhesins is the requirement of bile salt exposure for robust biofilm formation (29).

In addition to L. monocytogenes and S. flexneri, *Rickettsia* spp. and *Burkholderia* spp. display ABM in infected cells*. Burkholderia* spp. ABM is supported by the polar protein BimA (40). While Burkholderia thailandensis BimA activates the Arp2/3 complex, Burkholderia pseudomallei BimA facilitates actin nucleation and elongation by mimicking the nucleation activity of host cell Ena/VASP proteins (41). *Rickettsia* spp. exhibit early and late ABM phases driven by different surface proteins (42). Early ABM of *Rickettsia* spp. requires surface protein RickA, which stimulates Arp2/3 nucleation activity (43, 44). In the late ABM phase, the autotransporter Sca2 is needed for actin tail formation, independent of the Arp2/3 complex (42, 45), through molecular mimicry of host cell formin nucleation activity (46). It is unknown whether, as with ActA and IcsA, the ABM factors BimA, RickA, and Sca2 perform moonlighting functions. Interestingly, Sca2 promotes host cell adhesion and invasion when expressed in E. coli (47). However, the potential moonlighting adhesin functions of Sca2 have not been tested in *Rickettsia* spp.

How bacterial pathogens have evolved the ability to display ABM is a daunting question. The discovery of their moonlighting functions as discussed in this article may, however, offer some evolutionary perspectives. The protein sequence of ActA appears unique compared to existing sequences in publicly available databases, and the exact mechanisms supporting bacterial aggregation remain to be determined. ActA is a typical example of molecular mimicry, displaying short structural motifs that resemble motifs found in eukaryotic proteins (13). Whether these motifs have been acquired through convergent evolution or have been acquired from a eukaryotic protein through gene transfer remains an open debate. In contrast to the uniqueness of ActA, IcsA belongs to a large family of autotransporters whose passenger domain adopts an L-shaped β-helical structure that mediates self-association (35, 48). Most self-associating auto-transporters are produced by extracellular pathogens, and it is thus reasonable to assume that these factors do not bear intracellular ABM functions. Consequently, we propose that ABM factors have evolved from existing adhesins that were primordially dedicated to extracellular colonization of the host.

Our evolutionary perspectives predict the feasibility of genetically uncoupling ABM and adhesion functions. This task may be complex, as self-association properties of ABM factors are important for ABM efficiency (38, 49, 50). Structure/function analyses have so far suggested that disruption of ABM functions leads to disruption of adhesin functions (26, 37). However, the corresponding structure/function analyses relied on gross molecular lesions (deletions and insertions) that may have severely affected the scaffold of the ABM factors under investigation. Thus, identifying discrete mutations that specifically abrogate ABM functions but preserve ancestral adhesin functions, in a process that we refer to as “reverse evolution,” will constitute a critical endeavor for providing experimental support to the notion that ABM factors have evolved from ancestral adhesins.

Although functional novelties may arise neutrally in preexisting scaffolds, it has been proposed that some scaffolds may offer more flexibility in the evolution of novel functions in proteins, while maintaining ancestral functions (51). These scaffolds include disordered regions and loops in proteins. Interestingly, ActA has been shown to exist as a natively unfolded protein (49). Moreover, the predicted β-helical structure of autotransporters, such as Ag43 and IcsA, displays numerous nonstranded loops that may well accommodate substitutions in residues not essential for self-association (35, 48). Gene duplication is an important aspect of evolution that creates functional redundancy and opportunities for exploring mutational space, without jeopardizing ancestral functions. Eight of the nine trimeric auto-transporters present in B. pseudomallei enable bacterial adhesion to mammalian cells (52). Since these proteins appeared to play redundant roles, it is conceivable that mutational space was available for one of these proteins (BimA) to evolve ABM functions.

In conclusion, we speculate that, as extracellular pathogens evolved the ability to invade host cells and gain access to the cytosolic compartment, they encountered new selective pressures. In that context, we propose that ancestral extracellular adhesins coevolved through acquisition and fixation of discrete substitutions that conferred a selective advantage through creation of a novel intracellular function, ABM.

## References

[1] Jeffery CJ 1999 Moonlighting proteins. Trends Biochem Sci 24:8–11. doi:10.1016/S0968-0004(98)01335-8. 10087914

[2] Jeffery CJ 2018 Protein moonlighting: what is it, and why is it important? Philos Trans R Soc Lond B Biol Sci 373:20160523. doi:10.1098/rstb.2016.0523. 29203708 PMC5717523

[3] Rawat P, Kumar S, Sheokand N, Raje CI, Raje M 2012 The multifunctional glycolytic protein glyceraldehyde-3-phosphate dehydrogenase (GAPDH) is a novel macrophage lactoferrin receptor. Biochem Cell Biol 90:329–338. doi:10.1139/o11-058. 22292499

[4] Kumar S, Sheokand N, Mhadeshwar MA, Raje CI, Raje M 2012 Characterization of glyceraldehyde-3-phosphate dehydrogenase as a novel transferrin receptor. Int J Biochem Cell Biol 44:189–199. doi:10.1016/j.biocel.2011.10.016. 22062951

[5] Sheokand N, Malhotra H, Kumar S, Tillu VA, Chauhan AS, Raje CI, Raje M 2014 Moonlighting cell-surface GAPDH recruits apotransferrin to effect iron egress from mammalian cells. J Cell Sci 127:4279–4291. doi:10.1242/jcs.154005. 25074810

[6] Colell A, Green DR, Ricci J-E 2009 Novel roles for GAPDH in cell death and carcinogenesis. Cell Death Differ 16:1573. doi:10.1038/cdd.2009.137. 19779498

[7] Boël G, Jin H, Pancholi V 2005 Inhibition of cell surface export of group A streptococcal anchorless surface dehydrogenase affects bacterial adherence and antiphagocytic properties. Infect Immun 73:6237–6248. doi:10.1128/IAI.73.10.6237-6248.2005. 16177295 PMC1230963

[8] Jin H, Song YP, Boel G, Kochar J, Pancholi V 2005 Group A streptococcal surface GAPDH, SDH, recognizes uPAR/CD87 as its receptor on the human pharyngeal cell and mediates bacterial adherence to host cells. J Mol Biol 350:27–41. doi:10.1016/j.jmb.2005.04.063. 15922359

[9] Tunio SA, Oldfield NJ, Ala'Aldeen DAA, Wooldridge KG, Turner DP 2010 The role of glyceraldehyde 3-phosphate dehydrogenase (GapA-1) in Neisseria meningitidis adherence to human cells. BMC Microbiol 10:280. doi:10.1186/1471-2180-10-280. 21062461 PMC2994834

[10] Maeda K, Nagata H, Yamamoto Y, Tanaka M, Tanaka J, Minamino N, Shizukuishi S 2004 Glyceraldehyde-3-phosphate dehydrogenase of *Streptococcus oralis* functions as a coadhesin for *Porphyromonas gingivalis* major fimbriae. Infect Immun 72:1341–1348. doi:10.1128/IAI.72.3.1341-1348.2004. 14977937 PMC355992

[11] Querol-García J, Fernández FJ, Marin AV, Gómez S, Fullà D, Melchor-Tafur C, Franco-Hidalgo V, Albertí S, Juanhuix J, de Córdoba S, Regueiro JR, Vega MC 2017 Crystal structure of glyceraldehyde-3-phosphate dehydrogenase from the Gram-positive bacterial pathogen A. vaginae, an immunoevasive factor that interacts with the human C5a anaphylatoxin. Front Microbiol 8:541. doi:10.3389/fmicb.2017.00541. 28443070 PMC5385343

[12] Terao Y, Yamaguchi M, Hamada S, Kawabata S 2006 Multifunctional glyceraldehyde-3-phosphate dehydrogenase of Streptococcus pyogenes is essential for evasion from neutrophils. J Biol Chem 281:14215–14223. doi:10.1074/jbc.M513408200. 16565520

[13] Choe JE, Welch MD 2016 Actin-based motility of bacterial pathogens: mechanistic diversity and its impact on virulence. Pathog Dis 74:ftw099. doi:10.1093/femspd/ftw099. 27655913 PMC5968334

[14] Goldberg MB, Theriot JA 1995 Shigella flexneri surface protein IcsA is sufficient to direct actin-based motility. Proc Natl Acad Sci U S A 92:6572–6576. doi:10.1073/pnas.92.14.6572. 7604035 PMC41560

[15] Kocks C, Marchand J-B, Gouin E, d'Hauteville H, Sansonetti PJ, Carlier M-F, Cossart P 1995 The unrelated surface proteins ActA of Listeria monocytogenes and IcsA of Shigella flexneri are sufficient to confer actin-based motility on Listeria innocua and Escherichia coli respectively. Mol Microbiol 18:413–423. doi:10.1111/j.1365-2958.1995.mmi_18030413.x. 8748026

[16] Kuehl CJ, Dragoi AM, Talman A, Agaisse H 2015 Bacterial spread from cell to cell: beyond actin-based motility. Trends Microbiol 23:558–566. doi:10.1016/j.tim.2015.04.010. 26021574 PMC4560970

[17] Weddle E, Agaisse H 2018 Principles of intracellular bacterial pathogen spread from cell to cell. PLoS Pathog 14:e1007380. doi:10.1371/journal.ppat.1007380. 30543716 PMC6292572

[18] Kocks C, Gouin E, Tabouret M, Berche P, Ohayon H, Cossart P 1992 L. monocytogenes-induced actin assembly requires the actA gene product, a surface protein. Cell 68:521–531. doi:10.1016/0092-8674(92)90188-i. 1739966

[19] Welch MD, Iwamatsu A, Mitchison TJ 1997 Actin polymerization is induced by Arp 2/3 protein complex at the surface of Listeria monocytogenes. Nature 385:265–269. doi:10.1038/385265a0. 9000076

[20] Rafelski SM, Theriot JA 2006 Mechanism of polarization of Listeria monocytogenes surface protein ActA. Mol Microbiol 59:1262–1279. doi:10.1111/j.1365-2958.2006.05025.x. 16430699 PMC1413586

[21] Welch MD, Rosenblatt J, Skoble J, Portnoy DA, Mitchison TJ 1998 Interaction of human Arp2/3 complex and the *Listeria monocytogenes* ActA protein in actin filament nucleation. Science 281:105–108. doi:10.1126/science.281.5373.105. 9651243

[22] Skoble J, Portnoy DA, Welch MD 2000 Three regions within ActA promote Arp2/3 complex-mediated actin nucleation and *Listeria monocytogenes* motility. J Cell Biol 150:527–538. doi:10.1083/jcb.150.3.527. 10931865 PMC2175181

[23] Pistor S, Chakraborty T, Walter U, Wehland J 1995 The bacterial actin nucleator protein ActA of *Listeria monocytogenes* contains multiple binding sites for host microfilament proteins. Curr Biol 5:517–525. doi:10.1016/S0960-9822(95)00104-7. 7583101

[24] Pizarro-Cerdá J, Kühbacher A, Cossart P 2012 Entry of Listeria monocytogenes in mammalian epithelial cells: an updated view. Cold Spring Harb Perspect Med 2:a010009. doi:10.1101/cshperspect.a010009. 23125201 PMC3543101

[25] Suárez M, González-Zorn B, Vega Y, Chico-Calero I, Vázquez-Boland J-A 2001 A role for ActA in epithelial cell invasion by Listeria monocytogenes. Cell Microbiol 3:853–864. doi:10.1046/j.1462-5822.2001.00160.x. 11736996

[26] Travier L, Guadagnini S, Gouin E, Dufour A, Chenal-Francisque V, Cossart P, Olivo-Marin J-C, Ghigo J-M, Disson O, Lecuit M 2013 ActA promotes Listeria monocytogenes aggregation, intestinal colonization and carriage. PLoS Pathog 9:e1003131. doi:10.1371/journal.ppat.1003131. 23382675 PMC3561219

[27] Makino S, Sasakawa C, Kamata K, Kurata T, Yoshikawa M 1986 A genetic determinant required for continuous reinfection of adjacent cells on large plasmid in S. flexneri 2a. Cell 46:551–555. doi:10.1016/0092-8674(86)90880-9. 3524856

[28] Nickerson KP, Chanin RB, Sistrunk JR, Rasko DA, Fink PJ, Barry EM, Nataro JP, Faherty CS 2017 Analysis of Shigella flexneri resistance, biofilm formation, and transcriptional profile in response to bile salts. Infect Immun 85:e01067-16. doi:10.1128/IAI.01067-16. 28348056 PMC5442615

[29] Köseoğlu VK, Hall CP, Rodríguez-López EM, Agaisse H 2019 The autotransporter IcsA promotes *Shigella flexneri* biofilm formation in the presence of bile salts. Infect Immun 87:e00861-18. doi:10.1128/IAI.00861-18. 30988059 PMC6589070

[30] Drobnak I, Braselmann E, Chaney JL, Leyton DL, Bernstein HD, Lithgow T, Luirink J, Nataro JP, Clark PL 2015 Of linkers and autochaperones: an unambiguous nomenclature to identify common and uncommon themes for autotransporter secretion. Mol Microbiol 95:1–16. doi:10.1111/mmi.12838. 25345653 PMC4275399

[31] Goldberg MB, Barzu O, Parsot C, Sansonetti PJ 1993 Unipolar localization and ATPase activity of IcsA, a Shigella flexneri protein involved in intracellular movement. Infect Agents Dis 2:210–211. 8173795

[32] Charles M, Perez M, Kobil JH, Goldberg MB 2001 Polar targeting of Shigella virulence factor IcsA in Enterobacteriaceae and Vibrio. Proc Natl Acad Sci U S A 98:9871–9876. doi:10.1073/pnas.171310498. 11481451 PMC55545

[33] Egile C, Loisel TP, Laurent V, Li R, Pantaloni D, Sansonetti PJ, Carlier M-F 1999 Activation of the Cdc42 effector N-Wasp by the *Shigella flexneri* IcsA protein promotes actin nucleation by Arp2/3 complex and bacterial actin-based motility. J Cell Biol 146:1319–1332. doi:10.1083/jcb.146.6.1319. 10491394 PMC2156126

[34] Suzuki T, Miki H, Takenawa T, Sasakawa C 1998 Neural Wiskott-Aldrich syndrome protein is implicated in the actin‐based motility of *Shigella flexneri*. EMBO J 17:2767–2776. doi:10.1093/emboj/17.10.2767. 9582270 PMC1170617

[35] Mauricio RP, Jeffries CM, Svergun DI, Deane JE 2017 The Shigella virulence factor IcsA relieves N-WASP autoinhibition by displacing the verprolin homology/cofilin/acidic (VCA) domain. J Biol Chem 292:134–145. doi:10.1074/jbc.M116.758003. 27881679 PMC5217673

[36] May KL, Morona R 2008 Mutagenesis of the *Shigella flexneri* autotransporter IcsA reveals novel functional regions involved in IcsA biogenesis and recruitment of host neural Wiscott-Aldrich syndrome protein. J Bacteriol 190:4666–4676. doi:10.1128/JB.00093-08. 18456802 PMC2446779

[37] Brotcke Zumsteg A, Goosmann C, Brinkmann V, Morona R, Zychlinsky A 2014 IcsA is a Shigella flexneri adhesin regulated by the type III secretion system and required for pathogenesis. Cell Host Microbe 15:435–445. doi:10.1016/j.chom.2014.03.001. 24721572

[38] May KL, Grabowicz M, Polyak SW, Morona R 2012 Self-association of the Shigella flexneri IcsA autotransporter protein. Microbiology (Reading, Engl) 158:1874–1883. doi:10.1099/mic.0.056465-0. 22516224

[39] Klemm P, Vejborg RM, Sherlock O 2006 Self-associating autotransporters, SAATs: functional and structural similarities. Int J Med Microbiol 296:187–195. doi:10.1016/j.ijmm.2005.10.002. 16600681

[40] Stevens MP, Stevens JM, Jeng RL, Taylor LA, Wood MW, Hawes P, Monaghan P, Welch MD, Galyov EE 2005 Identification of a bacterial factor required for actin-based motility of Burkholderia pseudomallei. Mol Microbiol 56:40–53. doi:10.1111/j.1365-2958.2004.04528.x. 15773977

[41] Benanti EL, Nguyen CM, Welch MD 2015 Virulent Burkholderia species mimic host actin polymerases to drive actin-based motility. Cell 161:348–360. doi:10.1016/j.cell.2015.02.044. 25860613 PMC4393530

[42] Reed SCO, Lamason RL, Risca VI, Abernathy E, Welch MD 2014 Rickettsia Actin-based motility occurs in distinct phases mediated by different actin nucleators. Curr Biol 24:98–103. doi:10.1016/j.cub.2013.11.025. 24361066 PMC3951146

[43] Gouin E, Egile C, Dehoux P, Villiers V, Adams J, Gertler F, Li R, Cossart P 2004 The RickA protein of Rickettsia conorii activates the Arp2/3 complex. Nature 427:457. doi:10.1038/nature02318. 14749835

[44] Jeng RL, Goley ED, D’Alessio JA, Chaga OY, Svitkina TM, Borisy GG, Heinzen RA, Welch MD 2004 A Rickettsia WASP-like protein activates the Arp2/3 complex and mediates actin-based motility. Cell Microbiol 6:761–769. doi:10.1111/j.1462-5822.2004.00402.x. 15236643

[45] Kleba B, Clark TR, Lutter EI, Ellison DW, Hackstadt T 2010 Disruption of the *Rickettsia rickettsii* Sca2 autotransporter inhibits actin-based motility. Infect Immun 78:2240–2247. doi:10.1128/IAI.00100-10. 20194597 PMC2863521

[46] Haglund CM, Choe JE, Skau CT, Kovar DR, Welch MD 2010 Rickettsia Sca2 is a bacterial formin-like mediator of actin-based motility. Nat Cell Biol 12:1057–1063. doi:10.1038/ncb2109. 20972427 PMC3136050

[47] Cardwell MM, Martinez JJ 2009 The Sca2 autotransporter protein from Rickettsia conorii is sufficient to mediate adherence to and invasion of cultured mammalian cells. Infect Immun 77:5272–5280. doi:10.1128/IAI.00201-09. 19805531 PMC2786473

[48] Heras B, Totsika M, Peters KM, Paxman JJ, Gee CL, Jarrott RJ, Perugini MA, Whitten AE, Schembri MA 2014 The antigen 43 structure reveals a molecular Velcro-like mechanism of autotransporter-mediated bacterial clumping. Proc Natl Acad Sci U S A 111:457–462. doi:10.1073/pnas.1311592111. 24335802 PMC3890832

[49] Footer MJ, Lyo JK, Theriot JA 2008 Close packing of Listeria monocytogenes ActA, a natively unfolded protein, enhances F-actin assembly without dimerization. J Biol Chem 283:23852–23862. doi:10.1074/jbc.M803448200. 18577520 PMC2527104

[50] Mourrain P, Lasa I, Gautreau A, Gouin E, Pugsley A, Cossart P 1997 ActA is a dimer. Proc Natl Acad Sci U S A 94:10034–10039. doi:10.1073/pnas.94.19.10034. 9294158 PMC23296

[51] Copley SD 2014 An evolutionary perspective on protein moonlighting. Biochem Soc Trans 42:1684–1691. doi:10.1042/BST20140245. 25399590 PMC4405106

[52] Campos CG, Byrd MS, Cotter PA 2013 Functional characterization of Burkholderia pseudomallei trimeric autotransporters. Infect Immun 81:2788–2799. doi:10.1128/IAI.00526-13. 23716608 PMC3719557

